# Active Carboxymethylcellulose-Based Edible Films: Influence of Free and Encapsulated Curcumin on Films’ Properties

**DOI:** 10.3390/foods10071512

**Published:** 2021-06-30

**Authors:** Ana I. Bourbon, Maria J. Costa, Luís C. Maciel, Lorenzo Pastrana, António A. Vicente, Miguel A. Cerqueira

**Affiliations:** 1 International Iberian Nanotechnology Laboratory, Avenida Mestre José Veiga s/n, 4715-330 Braga, Portugal; ana.bourbon@inl.int (A.I.B.); maria.costa@inl.int (M.J.C.); lorenzo.pastrana@inl.int (L.P.); 2 Centre of Biological Engineering, Campus de Gualtar, University of Minho, 4710-057 Braga, Portugal; luiscbmaciel@gmail.com (L.C.M.); avicente@deb.uminho.pt (A.A.V.)

**Keywords:** active film, active coating, functional compounds, turmeric, nanoencapsulation

## Abstract

Carboxymethylcellulose (CMC)-based films can act as a protective barrier in food surfaces and a carrier of bioactive compounds, such as curcumin. However, incorporating curcumin in hydrophilic matrixes can be a challenge, and new strategies need to be explored. In this work, CMC-based films containing free curcumin and curcumin-loaded nanohydrogels (composed of lactoferrin and glycomacropeptide) were produced and characterized. The incorporation of curcumin-loaded nanohydrogels showed a significant decrease in films’ thickness (from 0.0791 to 0.029 mm). Furthermore, the water vapor permeability of CMC-based films was significantly decreased (62%) by incorporating curcumin-loaded nanohydrogels in the films. The water affinity’s properties (moisture, solubility, and contact angle) of films were also affected by incorporating encapsulated curcumin. The addition of nanohydrogels to CMC-based films reduced the tensile strength values from 16.46 to 9.87 MPa. Chemical interactions were analyzed using Fourier transform infrared spectroscopy. The release profile of curcumin from CMC-based films was evaluated at 25 °C using a hydrophilic food simulant and suggests that the release mechanism of the curcumin happens by Fick’s diffusion and Case II transport. Results showed that protein-based nanohydrogels can be a good strategy for incorporating curcumin in edible films, highlighting their potential for use in food applications.

## 1. Introduction

Films produced using renewable and edible materials present high potential as packaging materials and oral delivery carriers. They are considered a promising solution not only for environmental issues, but also due to their unique properties, which allow the controlled migration and release of several compounds, and have been proposed as carriers of functional compounds [1]. The protective barrier properties of the films and their ability to act as a carrier for bioactive compounds enhance the functional properties of foods, promote foods’ health benefits [2,3], and suggest potential use as oral delivery systems [4,5].

Several functional compounds with the potential to be used in the food industry are often poorly soluble in aqueous solutions, which represents a major barrier for their incorporation in matrixes and for efficient delivery. In contrast, the majority of edible films are produced using hydrophilic materials, in which hydrophobic functional compounds are difficult to incorporate. To overcome this issue, hydrophobic functional compounds can be encapsulated in micro- and nanoparticles, thus helping them disperse in film-forming solutions, and increasing their homogeneity and stability. When incorporated in edible films, these nanostructures can be used to control the release of several compounds, such as flavors, coloring, and antimicrobial substances, and can also be used to protect the active compounds from thermal and environmental conditions [6].

One of the compounds of interest is curcumin, which is present in the perennial herb *Curcuma longa* L. Curcumin is a polyphenol that shows several bioactive properties, such as antimicrobial, anti-inflammatory, and antioxidant properties, and also presents the ability to control the proliferation of a broad variety of tumor cells [7]. One main barrier to the bioavailability of curcumin is its poor water-solubility (hydrophobic) and fast metabolism [8]. Curcumin is soluble in ethanol, dimethyl sulfoxide, and acetone. Bourbon, Cerqueira and Vicente [9] developed protein-based nanohydrogels and showed their potential for the encapsulation of curcumin, aiming at the increase in their solubility and bioavailability.

Physical, chemical, and biological properties, and their form (free or entrapped) of active compounds, are major variables that interfere in the behavior of the active compounds in the film matrix. When entrapped in a structure, such as a particle or a fiber, their size (i.e., macro- to nanoscale) and chemical characteristics influence the film matrix and behavior of the active compound under different environmental conditions. Although edible films incorporated with functional compounds have been widely studied, the incorporation of curcumin and curcumin-loaded nanohydrogels into carboxymethylcellulose-based films, and the evaluation of their effect on films’ properties and the release behavior in a food simulant, is scarce [10,11,12,13]. Bojorges et al. [13] developed alginate films with turmeric that were applied to fresh beef, pork loin, and chicken breast for 12 days at 4 °C, and observed an antioxidant effect in meat, in addition to changes in color, and mechanical and barrier properties. Musso, Salgado and Mauri [12] prepared gelatin films with curcumin and concluded that curcumin affected the physicochemical properties of gelatin films. They observed that different colors could be obtained depending on the pH of the film-forming solution. In addition, antioxidant properties were observed and related to the presence of curcumin. Carboxymethylcellulose (CMC) is obtained from cellulose and is widely used in food, cosmetic, and pharmaceutical applications. CMC is an anionic polysaccharide and has been used in several edible film formulations due to its good film-forming ability and its non-toxicity, biodegradability, hydrophilicity, and biocompatibility properties [14].

This study aimed to evaluate the incorporation of free curcumin and curcumin-loaded nanohydrogels in CMC edible films, and to study their effect on physical and chemical properties and the release of curcumin in a food simulant.

## 2. Materials and Methods

### 2.1. Materials

Curcumin was purchased from Sigma-Aldrich (St. Louis, MO, USA). Sodium carboxymethylcellulose—CMC (Blanose, 7M65) was obtained from Ashland Inc. (Düsseldorf, Germany). Commercial glycomacropeptide (GMP) was kindly provided by Davisco Food International, Inc. (Le Sueur, MI, USA). Purified lactoferrin (Lf) was acquired in powder form from DMV International (Delhi, NY, USA) (iron content ≈ 120 ppm). Hydrochloric acid, absolute ethanol, and glycerol were obtained from Panreac (Barcelona, Spain).

### 2.2. Production of Nanohydrogels

The production of nanohydrogels and curcumin-loaded nanohydrogels was performed as presented in Bourbon et al. [9,15]. Nanohydrogels with a size around 112 ± 1.21 nm, a polydispersity index of 0.21 ± 0.04, and a zeta potential of −15.00 ± 0.75 mV were produced. The encapsulation efficiency of curcumin in Lf-GMP nanohydrogel is approximately 95.1 ± 1.4%.

### 2.3. Preparation of Carboxymethylcellulose Films

Carboxymethylcellulose (CMC) film-forming formulation was selected based on the previous study of Ramírez et al. [16]: 2 g of CMC were suspended in 100 mL of distilled water for 12 h at 25 °C under agitation; then, 1 g of glycerol was added to the solution and left for more 12 h under stirring.

Curcumin was added free and encapsulated in nanohydrogels to the film-forming solutions. Free curcumin (0.5 mg mL^−1^) was selected based on a preliminary evaluation that guaranteed a good distribution on films after drying. A quantity of 0.2% (*w/w*) of curcumin-loaded nanohydrogels (NG-BC) was added to film-forming solutions based on the previous work of Bourbon et al. [9] and preliminary experiments, aiming to achieve a good mix of the materials and processability of the films. This solution was cast into polystyrene Petri dishes, and dried for 48 h at 25 °C. Films were kept in desiccators at 20 °C and at a relative humidity (RH) of 53%.

### 2.4. Water Sensitivity

The water sensitivity of the films was analyzed through the determination of the moisture content, solubility, and water contact angle.

Moisture content was defined by the method described by Alves et al. [17], with some modifications. In brief, a circle of 50 mg of the film was used to perform these analyses.

To determine solubility, the dried samples (*Si*) were placed into cups with 50 mL of distilled water for 24 h at 150 rpm at room temperature (20 °C). The remaining insolubilized films were dried at 105 °C for 24 h and weighed (*Sf*). Solubility was calculated based on Equation (1):

(1) $$\mathrm{Solubility}\;\left(\%\right)=\left(Si-Sf\right)/Si\times 100$$

A face contact angle meter (OCA 20, Dataphysics, Germany) by the sessile drop method was used to determine the contact angle (*θ*) as described by Cerqueira et al. [18]. All measurements were performed on the face in contact with the air during film drying of the films at 22.2 ± 0.8 °C.

### 2.5. Water Vapor Permeability (WVP)

Water vapor permeability (WVP) was determined based on the work described by Costa et al. [19] using acrylic permeation cells produced in the Centre of Biological Engineering, Braga, Portugal. For each type of sample, three replicates were performed.

Film thickness was measured using a digital micrometer (No. 293-5, Mitutoyo, Kawasaki, Japan) with ±0.001 mm accuracy at ten different points.

### 2.6. Mechanical Properties

Mechanical properties of the films (elongation at break (EB) and tensile strength (TS)) were measured with an Instron Universal Testing Machine (Model 4500, Instron Corporation, Canton, OH, USA), following the methodology described by ASTM D882-10 with some modifications; samples were held between grips with 100 mm initial distance. Force and deformation variables were recorded during extension at 5 mm min^−1^. Five replicates (minimum) of each sample were executed.

### 2.7. Fourier Transform Infrared Spectroscopy (FTIR)

FTIR spectra of the films were analyzed using attenuated total reflection mode (ATR) in a Jasco Infrared Spectrometer (FT/IR-4100, Easton, MD, USA) device. A wavelength range between 4000 and 600 cm^−1^ at a resolution of 4 cm^−1^ was used. The absorbance of each FTIR spectrum was normalized between 0 and 1.

### 2.8. Opacity and Color

The color and opacity were defined as described in Costa et al. [20]. A Minolta colorimeter (Cr 400; Minolta, Tokyo, Japan) was used to determine the films’ color. A white standard color plate (Y = 93.9, x = 0.3158, y = 0.3321) was used as a background for the instruments’ calibration, for color measurements of the films. The opacity of the samples was determined according to the Hunter lab method (Equation (2)). The measurements were repeated five times for each film.

(2) $$\mathrm{Opacity}=\frac{Y_{b}}{Y_{w}}\;\times \;100$$

where *Y_b_* and *Y_w_* are the opacity of each sample on a black and white standard, respectively.

Five measurements were performed for each film sample.

### 2.9. Release Analysis

Films were cut into 4 cm^2^ square samples. These samples were immersed into a food simulant (for foods that have a hydrophilic character and can extract hydrophilic substances) with 50 mL of a 10% (*v/v*) ethanol solution, at 25 °C and 300 rpm. The kinetic release was determined by the evaluation of curcumin concentration release in solution until the equilibrium value was reached. The concentration of curcumin was assessed by measuring the absorbance (Elisa Biotech Synergy HT, Winooski, VT, USA) at 425 nm [9]. For each experimental condition, at least three replicates from the same film were conducted.

Release kinetics: The Berens and Hofenberg mathematical model [21] was fitted to experimental data of release profile of curcumin from films to evaluate the main release mechanisms involved during the release. The Berens and Hofenberg model accounts for two release mechanisms: the Fickian diffusion and the relaxation of the biopolymeric matrix:

(3) $$M_{t}{=M}_{t,F}{+M}_{t,\;R}$$

where *M_t,F_* represents the contributions of Fickian diffusion and *M_t,R_* represents the contributions of polymeric relaxations.

Fickian diffusion is described by the Brownian motion of molecules, and polymer relaxation is described by the swelling ability of polymer. Thus, the release of compounds from a hydrophilic polymer slab can be described by:

(4) $$M_{t}{=M}_{\infty ,F}\left[1-\frac{8}{\pi^{2}}\sum_{n=0}^{\infty }\frac{1}{{\left(2n+1\right)}^{2}}\exp[{\left(-2n+1\right)}^{2}{\;K}_{F}t]\right]+\sum_{i}M_{\infty ,Ri}\left[1\;-\exp\left({-K}_{Ri}t\right)\right]$$

where *K_F_* and *K_R_* are Fickian diffusion and relaxation rate constants, respectively.

The model of Equation (4) can then be used to describe pure Fickian (*M_1,F_* ≠ 0 and *i* = 0), anomalous (*M_1,F_* and *i*≠0), or Case II (*M_1,F_* = 0 and *i* ≠ 0) transport. Experimental results were analyzed by fitting Equation (4) (LSM) to assess the mechanisms involved in curcumin release from the films.

### 2.10. Statistical Analysis

Statistical analysis was completed using one-way ANOVA with a confidence level of 95% using Statistica^®^12 (Statsoft, Tulsa, OK, USA). To fit Equation (4) to experimental data the non-linear estimation module of Statistica^®^7 (Statsoft, Tulsa, OK, USA) was used. To determine the regression quality, the *R*^2^ coefficient and squared root mean square error (RMSE) were estimated, where the latter corresponds to the square root of the sum of the squared residues (SSE) divided by the regression degrees of freedom. Standardized halved width (SHW%) was also assessed to determine the precision of the estimated parameters.

## 3. Results and Discussion

**Figure 1:**
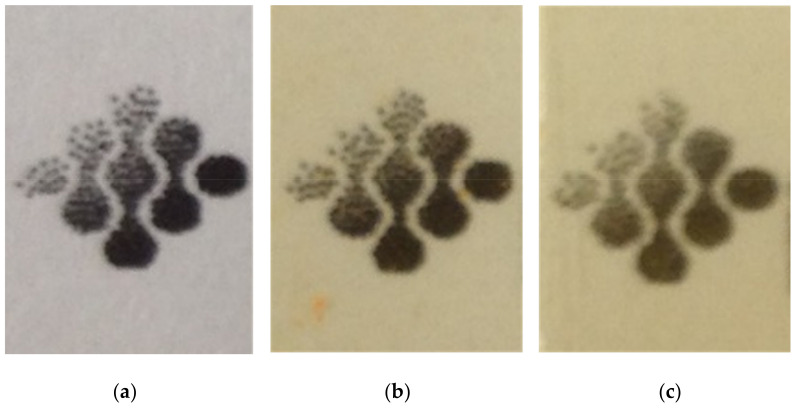
Images of the (**a**) CMC based-based film; (**b**) CMC-based film with free curcumin; (**c**) CMC-based film with curcumin-loaded nanohydrogels.

The effect on the film matrix of incorporating bioactive compounds depends on the physicochemical properties of the bioactive compounds (e.g., solubility) and biopolymer used (e.g., molecular weight and polarity). Moreover, when edible films are used as a carrier of bioactive compounds, the primary aim is to understand how the bioactive compounds will influence their processability and the final properties of the films. A preliminary evaluation was performed with the aim of incorporating the highest amount of curcumin into CMC-based films without affecting their processability; thus, 0.5 mg mL^−1^ of free curcumin and 0.2% (*w/v*) of curcumin-based nanohydrogel were selected (these values ensure a similar amount of curcumin in the film for free and encapsulated forms). Films obtained with these concentrations were homogeneous, flexible, and transparent, thus ensuring their further application as edible films (Figure 1).

### 3.1. Water Affinity

Moisture content, solubility, and surface hydrophobicity (contact angle) were determined to understand how the presence of curcumin in the free and encapsulated forms changes the film’s sensitivity to water.

Results show that the free curcumin decreases the water affinity of the films (Table 1).

The hydrophobic pattern of curcumin led to lower values of moisture content and solubility of the CMC-based films with free curcumin. In addition, the contact angle values showed that CMC-based films’ surfaces are more hydrophobic in ≈6% when curcumin is present. This behavior was observed in other studies, where curcumin increased the contact angle of CMC films [22], and can be explained by the hydrophobic characteristic of curcumin. Nevertheless, all of the films’ surfaces presented a hydrophilic behavior, with contact angle values lower than 60° [23], due to the hydrophilic nature of CMC [24].

In the case of CMC-based films with curcumin-loaded nanohydrogels, the values of solubility did not show a statistically significant difference (*p* > 0.05) to those of CMC-based films. It is possible that the encapsulation of curcumin into nanohydrogels avoids an influence of the curcumin regarding solubility properties of the CMC-based films because the hydrophobic part of curcumin is bonded to the hydrophobic part of the nanohydrogel [9]. In addition, the thickness of the CMC-based films with curcumin-loaded nanohydrogels (Table 1) is significantly lower than that of CMC-based films and CMC-based films with free curcumin, and thus influences the solubility values. This behavior may be due to the presence of nanohydrogels. The use of a protein can change the hydrophobic–hydrophilic balance, which can affect the interactions of the different materials (e.g., interaction with CMC and plasticizer). As a result, a decrease in the thickness of the CMC-based films with curcumin-loaded nanohydrogels was observed.

### 3.2. Water Vapour Permeability (WVP)

Table 1 presents the values of WVP of the studied films. The incorporation of free curcumin and curcumin-loaded nanohydrogels led to a decrease (*p* < 0.05) of the permeabilities values. For the CMC-based films with free curcumin, this decrease can be explained by the hydrophobic character of curcumin, which influenced the film matrix and decreased the diffusion of water vapor molecules through the film. Films with the curcumin-loaded nanohydrogels presented the lowest permeability values. The effect of nanosized structures in the film matrix is well studied; however, this effect is mostly described for nanoclays and nanocrystals, where the increase in tortuosity makes it difficult for the gas molecules to pass through the film matrix [25,26]. In addition, the change in the film matrix caused by the interaction between the CMC and the nanohydrogel can favor the decrease in the permeability, in which the gas molecules diffusion is decreased by the change in the free volume or density of the matrix. In this case, the incorporation of curcumin-loaded nanohydrogels decreased the permeabilities values by ≈3-fold for WVP.

These results are in agreement with the moisture content and solubility of the films, thus confirming the affinity of the films to water molecules (see Section 3.1). WVP values agree with previously reported values for CMC-based films [27,28]. Mirzaei-Mohkam et al. [1] nanoencapsulated vitamin E into CMC films and also observed a decrease of WVP values; this result was explained by the porosity caused by the nanocapsules of polycaprolactone, which led to a change in the CMC film matrix. Yai [29] encapsulated clove oil into hydroxypropyl methylcellulose-based films using chitosan to perform the encapsulation, and also observed a decrease in WVP due to the hydrophobic properties of the capsules. In addition, Li et al. [30] showed similar behavior with the incorporation if zein nanoparticles in sodium caseinate films, where they explained the behavior due to the hydrophobicity of the nanoparticles and the increase in tortuosity.

### 3.3. Mechanical Properties

Tensile strength (TS) and elongation-at-break (EB), in addition to providing useful information about the mechanical properties of the films, can be used to evaluate the effect of the materials added to the films’ structure. Table 1 presents the EB and TS values for the studied films, showing the effect of curcumin and curcumin-loaded nanohydrogels in CMC-based films. Both incorporation of free curcumin and curcumin-loaded nanohydrogels into CMC-based films led to a decrease (*p* < 0.05) in TS values of CMC films, which can be explained by their effect on the chemical structure of CMC-based films. The presence of the free curcumin and curcumin-loaded nanohydrogels decreases the intermolecular forces of the polysaccharide-based films resulting in a ductile material. The same behavior was observed by Roy and Rhim [22] with the incorporation of 1% curcumin in CMC films, which led to a decrease in the TS. This may be attributed to discontinuities in the polymer matrix and alterations in polymer chain interactions. Regarding EB, no effect was observed with the addition of free curcumin and curcumin-loaded nanohydrogels to CMC-based films, suggesting that the rigid properties of CMC were more prevalent than the elongation properties of the free curcumin and curcumin-loaded nanohydrogel in the films. The same behavior was observed by da Silva [31], who encapsulated treated curcumin (native curcumin treated using antisolvent precipitation) in hydroxypropyl methylcellulose.

### 3.4. Color and Opacity

Opacity and color parameters provide important information about the visual influence of incorporating bioactive compounds in edible films. Table 2 shows opacity values and color coordinates L* (lightness), a* (red/green), and b* (yellow/blue) for the CMC-based films, CMC-based films with free curcumin, and curcumin-loaded nanohydrogels.

Results showed that the film’s opacity increased for CMC-based films with free curcumin (8.45%) and CMC-based films with curcumin-loaded nanohydrogels (9.30%) when compared to CMC-based films (control) (5.01%). This was expected because the nanohydrogels and curcumin dispersion through the CMC matrix increases light scattering, leading to an increase in the opacity values [13]. Regarding color, the most significant change between films was observed for a* and b*, which was related to chromatic coordinates. As observed in Table 2, CMC-based films with curcumin-loaded nanohydrogels showed higher b* values compared to CMC-based films and CMC-based films with free curcumin, indicating a color transiting to yellowness (b* = 10.36), which was expected because of the curcumin color influence in these films [31]. These results can be confirmed visually in Figure 1.

### 3.5. Fourier Transform Infrared (FTIR) Spectroscopy

**Figure 2:**
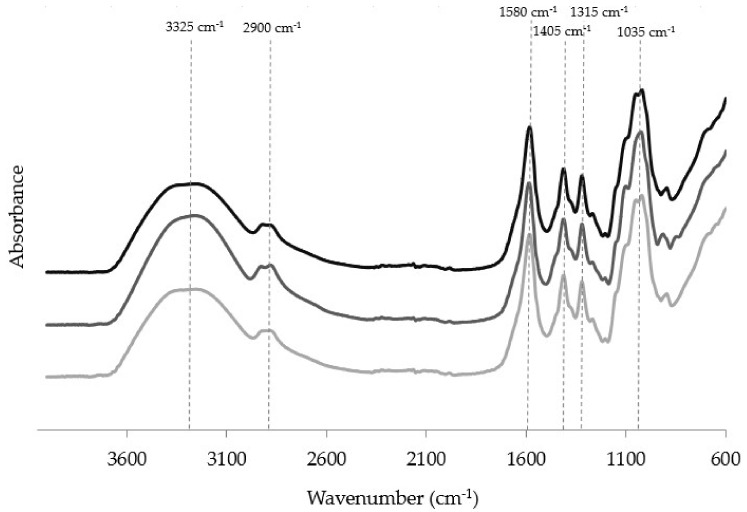
Fourier-transform infrared spectra obtained for the (**/**) CMC-based film; (**/**) CMC-based film with free curcumin; (**/**) CMC-based film with curcumin-loaded nanohydrogels.

The presence of curcumin and curcumin-loaded nanohydrogels in films was evaluated by FTIR to detect the characteristic peaks and possible physical bonds and chemical interactions between materials. FTIR spectra show the presence of typical peaks and bands of polysaccharide-based films (Figure 2); between 3600 and 3200 cm^−1^ there is a broad peak corresponding to –OH stretching vibrations; in the region between 3000 and 2800 cm^−1^, –CH stretching vibration is represented, and the carbohydrate region is expressed by the band in the region of 750–1300 cm^−1^[27]. The functional groups –C=O, –CH_2_, and –OH are represented at 1577, 1405 and 1315 cm^−1^ [28].

Free curcumin and curcumin-loaded nanohydrogels presence have different behaviors when added to the CMC-based films. The incorporation of curcumin in free form into the film matrix leads to the shift and the presence of new peaks. The peaks at 836 and 916 cm^−1^ correspond to the −CH stretching vibration of the aromatic ring of curcumin, which leads to a change in this region of CMC. At the wavenumber near 1025 cm^−1^, the stretching vibration of the aromatic ring and the bending of CH_3_ groups, which is typical of curcumin, leads to the change in the spectra of CMC-based films. In addition, a change in the –CH stretching vibration can be due to the presence of the –CH from the aromatic ring of curcumin [32].

For the CMC films with curcumin-loaded nanohydrogels, no differences were obtained when compared with CMC-based film spectra. The presence of curcumin encapsulated in the nanohydrogels leads to the absence of the characteristic peaks of curcumin, as observed in free curcumin in CMC-based films. This can be explained by the fact that curcumin is encapsulated in nanohydrogels and does not present the typical peaks. The same behavior was observed by other authors [33,34].

### 3.6. Release Experiments

**Figure 3:**
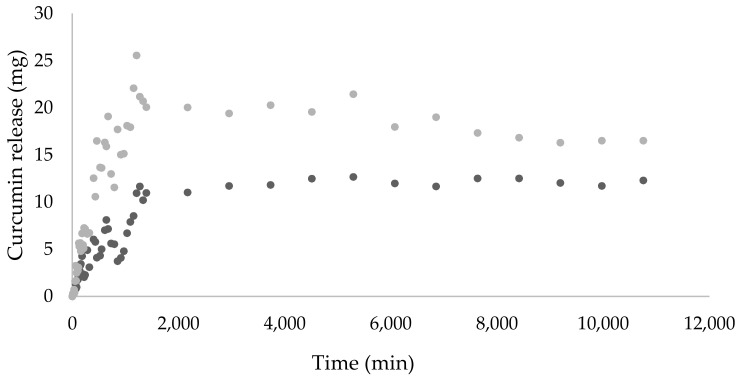
Release profile of free and encapsulated curcumin in CMC-based edible films in 10% ethanol at 25 °C: CMC-based film with free curcumin (●), CMC-based film with curcumin-loaded nanohydrogels (●).

The incorporation of nanostructures with bioactive compounds is a strategy to control the release of active molecules over time and to develop an active packaging. Thus, a release study was performed to evaluate the effect of protein nanohydrogels on curcumin release on CMC-based films. Figure 3 shows that the curcumin-loaded nanohydrogels CMC-based films have a higher release than the same amount of free curcumin CMC-based films.

This behavior suggests that curcumin molecules are chemically bonded to the CMC matrix, influencing their release. FTIR spectra (Figure 2) showed that after the encapsulation of curcumin in the CMC matrix, the peak with wavenumber near 1025 cm^−1^, typical of the aromatic ring and the bending of CH_3_ groups, was modified, suggesting a chemical modification, which agrees with the release profile.

**Figure 4:**
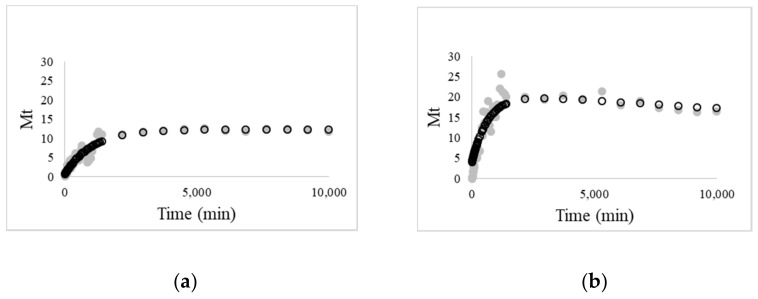
Linear superimposition model (LSM) description of curcumin release at 25 °C from: (**a**) CMC-based films with free curcumin and (**b**) CMC-based films curcumin-loaded nanohydrogels (experimental results (●); model-generated values (○)).

The description of the experimental data was performed using the linear superimposition model (Equation (4)). The selection of this model was based on its successful use to describe the release mechanisms of curcumin from biopolymeric matrices [9,35,36]. The fitting of the linear superimposition model to experimental data of curcumin released from CMC films and from protein nanohydrogels incorporated in CMC films, respectively, is presented in Figure 4.

Table 3 exhibits the regression analysis results of the LSM fitting for both compounds’ release, demonstrating that this model satisfactorily describes the experimental data with a reasonably good regression degree (*R*^2^ > 0.90) and that most of the parameters were estimated with good accuracy.

Analysis of the results from Table 3 shows that the release mechanism of the curcumin can only be described by both Fick’s diffusion and Case II transport. In both of the analyzed situations, it is possible to observe that the mass of curcumin released is mainly driven by the relaxation mechanism of the biopolymeric matrix, which is high for curcumin-loaded nanohydrogels. The behavior of the matrix (e.g., swelling, erosion) during the release of curcumin influences the total mass of curcumin released and, in the case of curcumin encapsulated in nanohydrogels, the relaxation rate is also higher than that of free curcumin in CMC-based films. This behavior reinforces the hypothesis that curcumin-loaded nanohydrogels are not chemically bonded to CMC-based films, as suggested by FTIR measurements, and therefore the release is higher.

## 4. Conclusions

This study shows that the incorporation of curcumin and curcumin-loaded nanohydrogels into carboxymethylcellulose-based films can affect the films’ properties and the release behavior of the active compound in a food simulant.

The incorporation of curcumin and curcumin-loaded nanohydrogels resulted in homogeneous, flexible, and transparent films. The solubility of CMC-edible films was influenced by curcumin incorporation, resulting in a significant decrease, whereas encapsulated curcumin did not affect the water affinity of the films. The water barrier permeability of the CMC-based films was also influenced by the incorporation of free curcumin and curcumin-loaded nanohydrogels, leading to a decrease (*p* < 0.05) in the permeability values. WVP was three-fold lower in CMC-edible films with curcumin-loaded nanohydrogels when compared to CMC-edible films. Release experiments in hydrophilic food simulants revealed that the curcumin-loaded nanohydrogel CMC-based films have a higher release compared to the same amount of free curcumin CMC-based films. In both of the analyzed situations, it was observed that the mass of curcumin released was mainly driven by the relaxation mechanism of the biopolymeric matrix, which was high for curcumin-loaded nanohydrogels. This work can contribute to establishing an approach to optimize the incorporation of hydrophobic bioactive compounds in edible films and their use in food systems with enhanced functionalities.

## Figures and Tables

**Table 1:** Film thickness, water sensitivity (moisture content, solubility, contact angle), water vapor permeability (WVP), tensile strength (TS), and elongation-at-break (EB) of CMC-based films, CMC-based films with free curcumin, and CMC-based films with curcumin-loaded nanohydrogels.

Film Samples	Thickness *×*10^−2^ (mm)	Moisture Content (%)	Solubility (%)	Contact Angle (°)	WVP × 10^−10^ (g·Pa^−1^·s^−1^·m^−1^)	TS (MPa)	EB (%)
CMC-based films	7.91 ^a^ (±1.23)	16.80 ^a^ (±1.74)	66.26 ^a^ (±4.17)	45.39 ^a^ (±3.70)	2.73 ^a^ (±0.08)	16.46 ^a^ (±2.83)	3.54 ^a^ (±1.48)
CMC-based films with free curcumin	6.99 ^a^ (±0.60)	13.18 ^b^ (±0.93)	46.12 ^b^ (±3.11)	49.93 ^b^ (±2.45)	2.22 ^b^ (±0.15)	11.23 ^b^ (±1.31)	4.19 ^a^ (±1.05)
CMC-based films with curcumin-loaded nanohydrogels	2.91 ^b^ (±0.39)	14.14 ^b^ (±2.68)	59.85 ^a^ (±9.78)	51.57 ^b^ (±2.52)	1.04 ^c^ (±0.08)	9.87 ^b^ (±1.18)	3.34 ^a^ (±0.84)

Values reported are the means ± standard deviations. Different letters (^a–c^) in the same column indicate a statistically significant difference (*p* < 0.05).

**Table 2:** Values of opacity, and L* (lightness), a* (red/green coordinate), and b* (yellow/blue coordinate) of CMC-based films, CMC-based films with free curcumin, and CMC-based films with curcumin-loaded nanohydrogels.

Film Samples	Opacity	L*	a*	b*
CMC-films	5.01 ^a^ (±0.98)	95.34 ^a^ (±1.52)	−0.69 ^a^ (±0.95)	3.50 ^a^ (±0.71)
CMC-films with free curcumin	8.45 ^b^ (±1.38)	96.85 ^b^ (±0.56)	−0.36 ^a^ (±0.12)	4.20 ^a^ (±0.65)
CMC-films with curcumin-loaded nanohydrogels	9.30 ^b^ (±0.14)	96.56 ^b^ (±0.13)	−2.33 ^b^ (±0.29)	10.36 ^b^ (±1.19)

Values reported are the means ± standard deviations. Different letters (^a,b^) in the same column indicate a statistically significant difference (*p* < 0.05).

**Table 3:** Results of fitting the linear superimposition model (LSM) (*i* = 1) to experimental data of curcumin release in CMC-based films with free curcumin and CMC-based films with curcumin-loaded nanohydrogels. Evaluation of the quality of the regression on the basis of RMSE and *R*^2^. Estimates’ precision is evaluated using the standardized halved width (SHW) % in parenthesis.

	RMSE	*R* ^2^	*M_F_*	*K_F_*	*M_R_*	*K_R_*
CMC-films with free curcumin	0.265	0.925	3.645 (27.85%)	9.579 (32.14%)	8.649 (47.35%)	5.124 (20.18%)
CMC-films with curcumin-loaded nanohydrogels	0.321	0.945	6.077 (57.35%)	14.845 (36.58%)	14.069 (19.87%)	16.941 (27.24%)

RMSE—Root mean square error. *M_F_*—Mass of compound released by Fickian transport. *K_F_*—Fickian diffusion rate constant. *M_R_*—Mass of compound released by relaxation of the matrix. *K_R_*—Relaxation rate constant.

## Data Availability

The data presented in this study are available on request from the corresponding author. The data are not publicly available due to privacy reasons.

## References

[1] Mirzaei-Mohkam A., Garavand F., Dehnad D., Keramat J., Nasirpour A. (2020). Physical, mechanical, thermal and structural characteristics of nanoencapsulated vitamin E loaded carboxymethyl cellulose films. Prog. Org. Coat..

[2] Silva-Weiss A., Ihl M., Sobral P.J.A., Gómez-Guillén M.C., Bifani V. (2013). Natural Additives in Bioactive Edible Films and Coatings: Functionality and Applications in Foods. Food Eng. Rev..

[3] Guimarães A., Abrunhosa L., Pastrana L.M., Cerqueira M.A. (2018). Edible Films and Coatings as Carriers of Living Microorganisms: A New Strategy Towards Biopreservation and Healthier Foods. Compr. Rev. Food Sci. Food Saf..

[4] Laffleur F., Keckeis V. (2020). Advances in drug delivery systems: Work in progress still needed?. Int. J. Pharm..

[5] Musazzi U.M., Khalid G.M., Selmin F., Minghetti P., Cilurzo F. (2020). Trends in the production methods of orodispersible films. Int. J. Pharm..

[6] González-Reza R.M., García-Betanzos C.I., Sánchez-Valdes L.I., Quintanar-Guerrero D., Cornejo-Villegas M.A., Zambrano-Zaragoza M.L. (2018). The functionalization of nanostructures and their potential applications in edible coatings. Coatings.

[7] Wang Y., Lu J.U.N., Jiang B., Guo J. (2020). The roles of curcumin in regulating the tumor immunosuppressive microenvironment (Review). Oncol. Lett..

[8] Pathak L., Amrutanand T., Agrawal Y. (2017). Alginate-chitosan coated lecithin core shell nanoparticles for curcumin: Effect of surface charge on release properties and biological activities. Indian J. Pharm. Educ. Res..

[9] Bourbon A.I., Cerqueira M.A., Vicente A.A. (2016). Encapsulation and controlled release of bioactive compounds in lactoferrin-glycomacropeptide nanohydrogels: Curcumin and caffeine as model compounds. J. Food Eng..

[10] Rachtanapun P., Klunklin W., Jantrawut P., Jantanasakulwong K., Phimolsiripol Y., Seesuriyachan P., Leksawasdi N., Chaiyaso T., Reungsang A., Ngo T. (2021). Characterization of Chitosan Film Incorporated with Curcumin Extract. Polymers.

[11] Chen L., Song Z., Zhi X., Du B. (2020). Photoinduced Antimicrobial Activity of Curcumin-Containing Coatings: Molecular Interaction, Stability and Potential Application in Food Decontamination. ACS Omega.

[12] Musso Y.S., Salgado P.R., Mauri A.N. (2017). Food Hydrocolloids Smart edible films based on gelatin and curcumin. Food Hydrocoll..

[13] Bojorges H., Ríos-Corripio M.A., Hernandéz-Cázares A.S., Hidalgo-Contreras J.V., Contreras-Oliva A. (2020). Effect of the application of an edible film with turmeric (*Curcuma longa* L.) on the oxidative stability of meat. Food Sci. Nutr..

[14] Singh P., Magalhães S., Alves L., Antunes F., Miguel M., Lindman B., Medronho B. (2019). Cellulose-based edible films for probiotic entrapment. Food Hydrocoll..

[15] Bourbon A.I., Pinheiro A.C., Cerqueira M.A., Vicente A.A. (2018). In vitro digestion of lactoferrin-glycomacropeptide nanohydrogels incorporating bioactive compounds: Effect of a chitosan coating. Food Hydrocoll..

[16] Ramírez C., Gallegos I., Ihl M., Bifani V. (2012). Study of contact angle, wettability and water vapor permeability in carboxymethylcellulose (CMC) based film with murta leaves (Ugni molinae Turcz) extract. J. Food Eng..

[17] Alves D., Cerqueira M.A., Pastrana L.M., Sillankorva S. (2020). Entrapment of a phage cocktail and cinnamaldehyde on sodium alginate emulsion-based films to fight food contamination by Escherichia coli and Salmonella Enteritidis. Food Res. Int..

[18] Cerqueira M.A., Costa M.J., Fuciños C., Pastrana L.M., Vicente A.A. (2013). Development of Active and Nanotechnology-based Smart Edible Packaging Systems: Physical–chemical Characterization. Food Bioprocess Technol..

[19] Costa M.J., Marques A.M., Pastrana L.M., Teixeira J.A., Sillankorva S.M., Cerqueira M.A. (2018). Physicochemical properties of alginate-based films: Effect of ionic crosslinking and mannuronic and guluronic acid ratio. Food Hydrocoll..

[20] Costa M.J., Pastrana L.M., Teixeira J.A., Sillankorva S.M., Cerqueira M.A. (2021). Characterization of PHBV films loaded with FO1 bacteriophage using polyvinyl alcohol-based nanofibers and coatings: A comparative study. Innov. Food Sci. Emerg. Technol..

[21] Berens A.R., Hopfenberg H.B. (1978). Diffusion and relaxation in glassy polymer powders: 2. Separation of diffusion and relaxation parameters. Polymer.

[22] Roy S., Rhim J.W. (2020). Carboxymethyl cellulose-based antioxidant and antimicrobial active packaging film incorporated with curcumin and zinc oxide. Int. J. Biol. Macromol..

[23] Cherpinski A., Jari S.T., Maria V., Peresin S., Lahtinen P., Lagaron J.M. (2018). Improving the water resistance of nanocellulose-based films with polyhydroxyalkanoates processed by the electrospinning coating technique. Cellulose.

[24] Simsek M., Eke B., Demir H. (2020). Characterization of carboxymethyl cellulose-based antimicrobial films incorporated with plant essential oils. Int. J. Biol. Macromol..

[25] Mariano M., Kissi N.E., Dufresne A. (2014). Cellulose Nanocrystals and Related Nanocomposites: Review of some Properties and Challenges. J. Polym. Sci. Part B Polym. Phys..

[26] Martins J.T., Bourbon A.I., Pinheiro A.C., Souza B.W.S., Cerqueira M.A., Vicente A.A. (2013). Biocomposite Films Based on κ-Carrageenan/Locust Bean Gum Blends and Clays: Physical and Antimicrobial Properties. Food Bioprocess Technol..

[27] Ballesteros L.F., Cerqueira M.A., Teixeira J.A., Mussatto S.I. (2018). Production and physicochemical properties of carboxymethyl cellulose films enriched with spent coffee grounds polysaccharides. Int. J. Biol. Macromol..

[28] Michelin M., Marques A.M., Pastrana L.M., Teixeira A. (2020). Carboxymethyl cellulose-based films : Effect of organosolv lignin incorporation on physicochemical and antioxidant properties. J. Food Eng..

[29] Yai H. (2011). Antimicrobial activity and the properties of edible hydroxypropyl methylcellulose based films incorporated with encapsulated clove (Eugenia caryophyllata Thunb.) oil. Int. Food Res. J..

[30] Li K., Yin S., Yang X., Tang C., Wei Z. (2012). Fabrication and Characterization of Novel Antimicrobial Films Derived from Thymol-Loaded Zein − Sodium Caseinate (SC) Nanoparticles. J. Agric. Food Chem..

[31] da Silva M.N., de Matos Fonseca J., Feldhaus H.K., Soares L.S., Valencia G.A., Maduro de Campos C.E., Di Luccio M., Monteiro A.R. (2019). Physical and morphological properties of hydroxypropyl methylcellulose films with curcumin polymorphs. Food Hydrocoll..

[32] Kolev T.M., Velcheva E.A., Stamboliyska B.A., Spiteller M. (2005). DFT and experimental studies of the structure and vibrational spectra of curcumin. Int. J. Quantum Chem..

[33] Das R.K., Kasoju N., Bora U. (2010). Encapsulation of curcumin in alginate-chitosan-pluronic composite nanoparticles for delivery to cancer cells. Nanomed. Nanotechnol. Biol. Med..

[34] Rocha B., Gonçalves O., Leimann F., Rebecca E., Silva-Buzanello R., Filho L., Araújo P., Cuman R., Bersani-Amado C. (2014). Curcumin encapsulated in poly-L-lactic acid improves its anti-inflammatory efficacy in vivo. Adv. Med. Plant Res..

[35] Pinheiro A.C., Bourbon A.I., Quintas M.A.C., Coimbra M.A., Vicente A.A. (2012). Κ-Carrageenan/Chitosan Nanolayered Coating for Controlled Release of a Model Bioactive Compound. Innov. Food Sci. Emerg. Technol..

[36] Pinheiro A.C., Bourbon A.I., Vicente A.A., Quintas M.A.C. (2013). Transport mechanism of macromolecules on hydrophilic bio-polymeric matrices—Diffusion of protein-based compounds from chitosan films. J. Food Eng..

